# Pre‐mRNA splicing is modulated by antifungal drugs in the filamentous fungus *Neurospora crassa*

**DOI:** 10.1002/2211-5463.12047

**Published:** 2016-03-14

**Authors:** Niege S. Mendes, Patricia M. Silva, Rafael Silva‐Rocha, Nilce M. Martinez‐Rossi, Antonio Rossi

**Affiliations:** ^1^ Department of GeneticsRibeirão Preto Medical SchoolUniversity of São PauloRibeirão PretoSPBrazil; ^2^ Department of Molecular and Cellular BiologyRibeirão Preto Medical SchoolUniversity of São PauloRibeirão PretoSPBrazil

**Keywords:** alternative splicing, amphotericin B, asparagine synthetase, intron retention, ketoconazole, *Neurospora crassa*, Pi regulation

## Abstract

For this study, we sought to identify pre‐mRNA processing events modulated by changes in extracellular pH, inorganic phosphate, and antifungal drugs. We examined genes with at least four putative introns whose transcriptional level responded to these effectors. We showed that the intron retention levels of genes encoding asparagine synthetase 2, C6‐zinc finger regulator (*fluffy*), and a farnesyltransferase respond to amphotericin B, ketoconazole, and other effectors. In general, the assayed antifungals promoted the disruption of the structural domains of these proteins probably leading to their inactivation, which emphasize the complexity of the metabolic modulation exerted by antifungal signaling.

**Abbreviations**
MAPK: mitogen‐activated protein kinase
*nuc‐1*: transcriptional activator which controls phosphate acquisition
*nuc‐2*: component of Pi‐regulated signal transduction pathway
Pi: phosphate
*preg* and *pgov*: regulatory genes which control phosphate acquisition
qRT‐PCR: quantitative real‐time PCR
RNAseq: next‐generation RNA sequencing technologies
SRA: Sequence Read Archive
TCA: tricarboxylic acid

Alternative splicing of pre‐mRNA transcripts is a regulated and complex molecular process that dramatically increases proteome diversity and fulfills important post‐transcriptional regulatory functions 1, 2, 3, 4, 5, 6, 7. There are at least five types of alternative splicing in eukaryotic microorganisms that are represented by alternate 5′ or 3′ splice sites, exon skipping, intron retention, and mutually exclusive events 2, 8, 9, 10. In filamentous fungi, the splicing of numerous genes is modulated in response to nutrient signaling. These include, for example, the genes *hex*‐1 of *Neurospora crassa* and *pacC* of *Aspergillus nidulans*, which respond to extracellular phosphate changes and ambient pH, respectively 11, 12, 13.

The filamentous fungus *N. crassa* is a heterothallic, free‐living, eukaryotic microorganism that is widespread in nature and is clearly visible due to its intense pink color. The importance of *N. crassa* in biochemical and molecular genetics research has been long recognized, making it an excellent model system for examining molecular responses to current signals in eukaryotic microorganisms 14, 15. Ambient signals are first detected by sensors, which then initiate the transmission of intracellular information via transduction pathways to a target 16, 17, 18, 19, 20. Changes in ambient temperature, light, pH, nutrients, and the presence of fungicides are among the extensive repertoire of fungal stressors that are detectable.

Phosphate (Pi) is a crucial ingredient in the synthesis of nucleic acids and the flow of genetic information 21, 22. In *N. crassa*, the molecular response to Pi deprivation consists of a highly conserved and hierarchical relationship among at least five genes—*nuc‐2*,* preg*,* pgov*,* mak‐2*, and *nuc‐1*—whose functioning allows for the activation of the transcriptional regulator NUC‐1 and its translocation into the nucleus. This translocation, in turn, leads to the transcriptional activation of several genes, including the Pi‐repressible phosphatases 15, 19, 23, 24, 25, 26, 27, 28, 29. Previously published microarray experiments using a *mak‐2* knockout strain (Δ*mak‐2*) confirmed that the *mak‐2* gene, which encodes a mitogen‐activated protein kinase (MAPK), is an important component of the response to extracellular Pi changes in *N. crassa* 19. These experiments revealed many differentially expressed genes that are involved in various physiological processes related to Pi transport, metabolism, and regulation, as well as to post‐translational modification of proteins and the MAPK MAK‐2 signaling pathway. Among them are some genes that are not apparently related to Pi scavenging, such as the genes encoding asparagine synthetase 2 (KEGG: NCU04303) 19, 30, 31, 32, C6‐zinc finger regulator (*fluffy*, KEGG: NCU08726) 33, 34, 35, and a farnesyltransferase (KEGG: NCU05999) 36, 37. Several genes that are repressed in the wild‐type strain when grown in the presence of high Pi, such as *nuc‐2*, are repressed in the Δ*mak‐2* mutant strain when grown in either low‐ or high‐Pi media. In an extended model of the Pi‐signaling network, we proposed that the MAK‐2 and NUC‐2 proteins are functional in *N. crassa* that has been cultured under conditions of limited Pi, but are nonfunctional under sufficient Pi conditions, leading to a reduction in the transcription of Pi‐repressible genes. Here, we show that intron retention by the genes coding for asparagine synthetase 2 (*asn‐2*), C6‐zinc finger regulator (*fluffy*), and a farnesyltransferase (*ram‐1*) occurs in response to extracellular pH and Pi changes and the presence of antifungal drugs.

## Materials and methods

### Strains, culture conditions, disk diffusion assay, and cDNA synthesis

The *N. crassa* wild‐type St.L.74‐OR23‐1VA (FGSC No 2489) (control strain), and the Δ*mak‐2* mutant strain (FGSC No 11482) of *N. crassa* used in this study were obtained from the Fungal Genetics Stock Center, Kansas State University, Manhattan, Kansas 38 (www.fgsc.net). The Δ*mak‐2* mutant strain was generated by the *Neurospora Functional Genomics Project Strains* (www.fgsc.net). The strains were maintained on slants of Vogel's medium (1.5% agar, 2% sucrose).

The *in vitro* susceptibilities of two *N. crassa* strains (St.L.74A and Δ*mak*‐2) to the antifungal drugs ketoconazole, amphotericin B, nystatin, and terbinafine were evaluated using a disk diffusion assay at pH 5.4 39, 40, 41. After spreading aliquots of 200 μL conidial suspension containing about 2 × 10^6^ cells·mL^−1^ of each strain on the agar plate surface, filter paper disks (5‐mm diameter) containing different quantities of each antifungal drug were placed on the center of the plates. All fungi were tested with three biological replicates. Plates were incubated for 72 h at 30 °C. Inhibitor zones for the experiments were measured after visible fungal lawns had covered the control plates. These were plotted against the antifungal concentration to determine the standard deviation for the inhibition zone (< 10% for all experiments) and to define the antifungal concentrations used in gene expression assays.

For gene expression assays, conidia (approximately 10^6^ cells·mL^−1^) were germinated for 5 h and 16 h at 30 °C in an orbital shaker (200 rpm) in low‐ (100 μm) or high‐Pi (10 mm) medium. The medium was adjusted to either pH 5.4 or 8.0 and was supplemented with 44 mm sucrose as the carbon source. Cells were grown in the presence or absence of amphotericin B (200 μg·mL^−1^) or ketoconazole (1 mg·mL^−1^) and prepared as previously described 42, 43.

Total RNA was extracted from approximately 100 mg of frozen mycelia using the Illustra RNAspin mini isolation kit (GE Healthcare, Little Chalfont, UK) and treated with RNAse‐free DNAse I (Invitrogen, Carlsbad, CA, USA). Purified RNA (1 μg) from each strain was reverse‐transcribed into cDNA with the High Capacity cDNA Reverse Transcription Kit (Applied Biosystems, Foster City, CA, USA) according to the manufacturer's instructions. The absence of genomic DNA in RNA preparations was confirmed by RT‐PCR using a primer set targeting an intron‐flanking region in the *N. crassa* actin gene.

### RNA‐seq quantification of intron retention events

Publicly available RNAseq data (next‐generation RNA sequencing technologies) from the Sequence Read Archive at the NCBI database (http://www.ncbi.nlm.nih.gov/sra) was used to quantify intron retention events in *N. crassa*. Seven RNAseq experiments for *N. crassa* wild‐type strains grown on glucose (GEO: GSM1238604 and GSM1238606), xylose (GEO: GSM1238609), arabinose (GEO: GSM1238602 and GSM1238597), sucrose (GEO: GSM899613), and cellulose (Avicel, GEO: GSM899607) were used. For intron retention quantification, SRA files were downloaded from the database and converted to *fastq* format using the SRA toolkit. Reads were then aligned to *fasta* files containing sequences of 50 nt representing each exon–intron boundary for the genes of interest (KEGG: NCU04303, NCU08726, and NCU05999) using Bowtie 44, 45. The number of reads mapped at each boundary was used to estimate the prevalence of intron retention events in each gene.

### Qualitative and quantitative real‐time PCR (qRT‐PCR)

For qualitative expression analysis, primer pairs that yield PCR products surrounding each intron sequence (Table 1) were used to amplify the transcription products of the genes coding for asparagine synthetase 2 (KEGG: NCU04303), C6‐zinc finger regulator (KEGG: NCU08726), and a farnesyltransferase (KEGG: NCU05999). For each PCR reaction, we used approximately 100 ng of cDNA and 10 pmol of each oligonucleotide. Thermocycler conditions were 95 °C for 2 min, followed by 35 cycles at 95 °C for 30 s with an annealing temperature of 60 °C for 1 min. Only relevant results are shown in the figures.

**Table 1:** Primers used in RT‐PCR and/or qRT‐PCR experiments

Gene product name (gene name)	ID^a^	Primer sequences (5′‐3′)	Amplicon length (bp)	Amplicon spliced (bp)	Intron analyzed	Experiments
Asparagine synthetase 2 (*asn‐2*)	NCU04303					
	F:	ATCCGTCACCGTGGTCCTGAT	188	49	Intron 3	RT‐PCR
	R:	TGTTGTGGCAAGTGACGCTGC				
	F:	CAACCTTTCGCCCAACC	63	–	Intron 3	qRT‐PCR
	R:	TTGACTCGATTGACACTTTTAT				
	F:	GCGGCAGCGTCACTTG	142	57	Intron 4	RT‐PCR
	R:	CCGACGATACTGAGACGCT				
	F:	TGCGGATAAGACGATGGTTG	182	112	Intron 5	RT‐PCR
	R:	TGCCAAGCGTCTTGTTTCTA				
C6‐zinc finger (*fluffy*)	NCU08726					
	F:	GCCAAGACAACACCTAACACCGAA	299	149	Intron 1	RT‐PCR
	R:	AGATGGTCCTTGGTACGCCATTTC				
CaaX farnesyltransferase beta subunit Ram1 (*ram‐1*)	NCU05999					
	F:	CAACCCAACAACGAGGCCCATGGC	503	313	Intron 3	RT‐PCR
	R:	CGACTATAGAGACTCTCCGGCGCA				
Actin	NCU04173					
	F:	GTATGTGCAAGGCCGGTTTCG	305	109	Introns 3/4	RT‐PCR qRT‐PCR
	R:	TCCTTCTGGCCCATACCGATCATG				
Tubulin alpha‐1	NCU09132					
	F:	CCTCGTCTTCCACTCCTTCGG	445	190	Intron 4	RT‐PCR qRT‐PCR
	R:	ACTGTGTTCGAGGGTGCTGTG				

a Gene accession number at the *N. crassa* genome database at the Broad Institute.

Quantitative real‐time PCR amplifications of *NCU04303 (asparagine synthetase 2)* were performed with the StepOne Plus Real‐Time PCR system (Applied Biosystems) using the oligonucleotides in Table 1. The qPCR experiments were performed in a 12.5‐μL reaction containing 6.25‐μL of the SYBR Green PCR Master Mix (Applied Biosystems), 50 ηg of cDNA, and 1 μL of each primer. The PCR protocol included an initial denaturation step at 50 °C for 2 min and at 95 °C for 10 min, followed by 40 cycles of 95 °C for 15 s and 60 °C for 1 min. A dissociation curve was generated at the end of each PCR cycle to verify the amplification of a single product. Relative transcript quantities were calculated using the ΔΔ*C*_t_ method 11, 46 with *N. crassa actin* and *β‐tubulin* as the internal reference genes. Analysis was performed using the onestep software v2.2 (Applied Biosystems, Foster City, CA, USA). The control strain grown in high‐Pi conditions, pH 5.4 for 5 h and in the absence of antifungal was used as a reference for calculating relative gene expression. Statistical analysis was performed using one‐way ANOVA followed by the Tukey's *ad hoc* test using graphpad prism v5.1 software (La Jolla, CA, USA).

## Results and Discussion

Previously, genome‐wide differential transcription profiling using a Δ*mak‐2* knockout strain of *N. crassa* grown under phosphate shortage revealed 912 unique genes that were found to be differentially expressed when compared to the expression in the control strain (S.t.L.74A) 19. Functional annotation of those differentially expressed genes using the Gene Ontology 47, 48 and *N. crassa* Genome Database 49, 50 showed that these genes were primarily involved in various physiological processes related to phosphate transport, metabolism, and regulation; posttranscriptional modification of proteins; and MAPK signaling pathways. In order to identify alternative splicing events in the transcriptional processing of these 912 genes we preselected 13 of them, which met the criteria of having at least four putative introns and being responsive to changes in ambient Pi or a *mak‐2* ^−^ background 19. Qualitative expression analyses by RT‐PCR identified three candidate genes coding for asparagine synthetase 2 (*asn‐2*) (KEGG: NCU04303), C‐6‐zinc finger (*fluffy*) (KEGG: *NCU08726*), and CaaX farnesyl transferase beta subunit Ram1 (*ram‐1*) (KEGG: NCU05999) for further study (Table 1).

**Figure 1:**
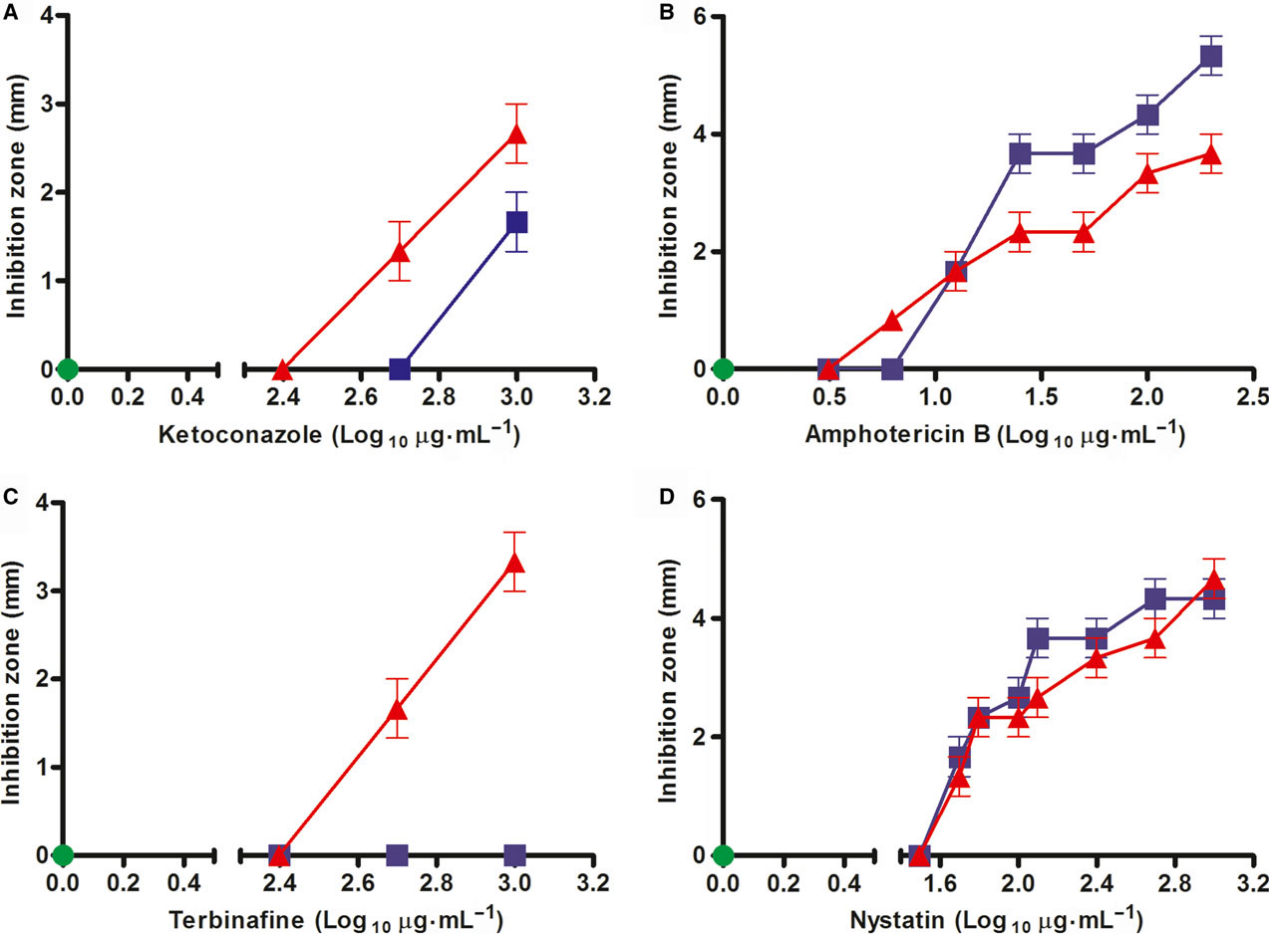
*In vitro* susceptibility of two *N. crassa* strains exposed to antifungal drugs at pH 5.4. The inhibition zones, measured in mm, were plotted against antifungal concentrations. The St.L.74A and Δ*mak‐2* strains were incubated in the absence of antifungal (control) (green circles). Strains were also incubated with ketoconazole (A), amphotericin B (B), terbinafine (C), and nystatin (D). Blue squares and red triangles represent the inhibition zones observed for the St.L.74A and Δ*mak‐2* strains, respectively.

Assays assessing the *in vitro* susceptibility of *N. crassa* to some antifungal drugs showed that the Δ*mak‐2* knockout strain is more susceptible to ketoconazole and terbinafine than the wild‐type strain. In contrast, the Δ*mak‐2* strain is more tolerant to amphotericin B but has the same tolerance to nystatin (Fig. 1). These results suggest that MAK‐2 affects the inhibitory mechanism and the sensing of these antifungal drugs. Ketoconazole and amphotericin B were therefore selected for our intron retention assays because they presented opposite effects in a comparison of the wild‐type strain with the Δ*mak‐2* strain. Furthermore, the control strain is not affected by terbinafine at the concentrations used (Fig. 1).

**Figure 2:**
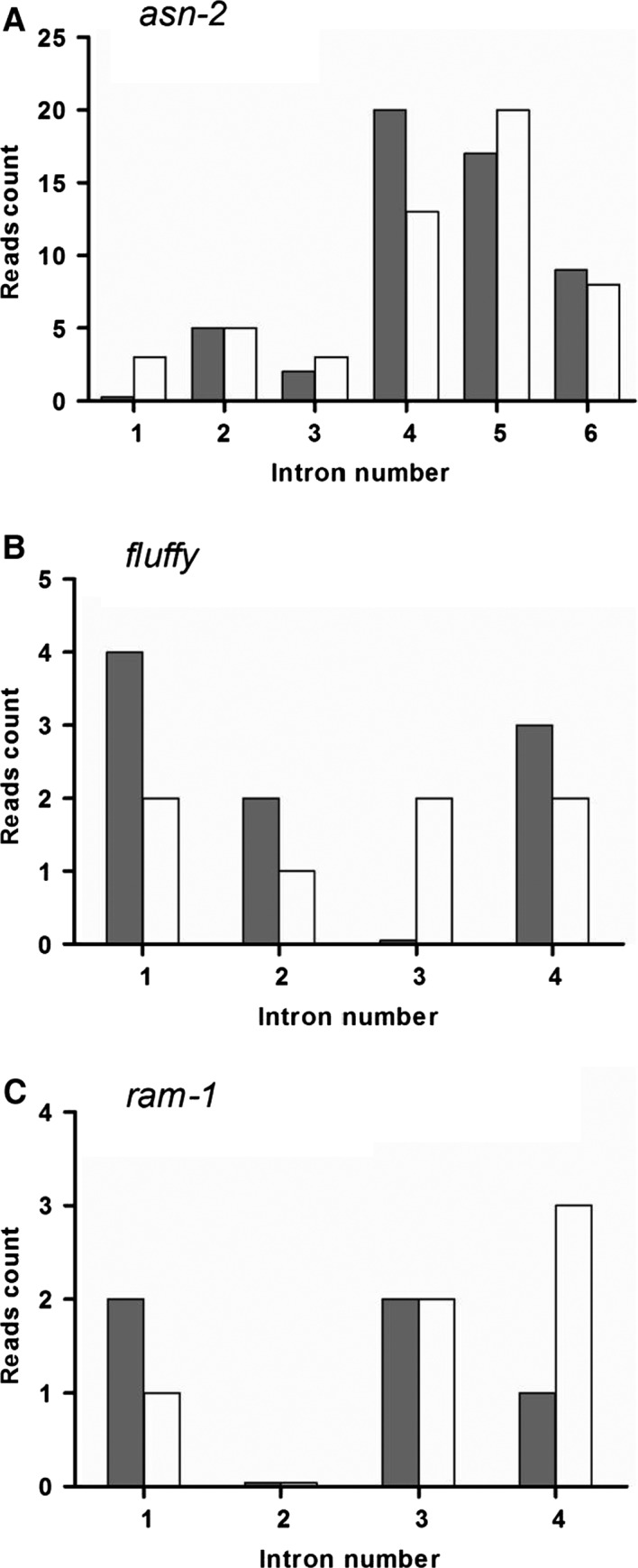
Intron retention evaluated by RNAseq in *N. crassa*. The Sequence Read Archive at the NCBI database (http://www.ncbi.nlm.nih.gov/sra) was used to quantify intron retention. Reads were then aligned to *fasta* files containing sequences of 50 nt representing each exon‐intron boundary for the genes of interest. The number of reads mapping at each boundary was used to estimate the prevalence of intron retention in each gene analyzed (5′‐ gray; 3′‐ white). (A) Asparagine synthetase 2 (*asn‐2*) (NCU04303); (B) C6‐zinc finger regulator (*fluffy*) (NCU08726); (C) farnesyltransferase (*ram1*) (NCU05999).

**Figure 3:**
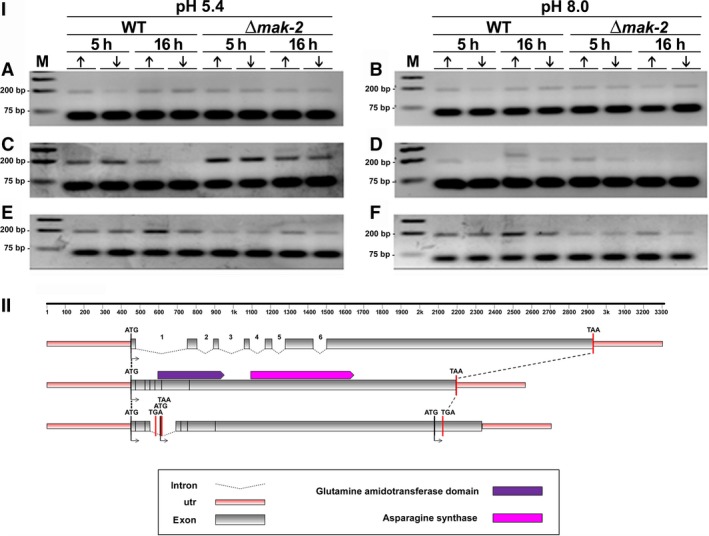
(I) Retention of intron‐3 visualized by RT‐PCR during pre‐mRNA processing of the *asn‐2* gene in *N. crassa*. Strains Δ*mak‐2* mutant and St.L.74A were incubated for 5 h and 16 h in high‐ (10 mm) (↑) or low‐Pi (100 μm) (↓) at pH 5.4 and pH 8.0 in the absence of antifungal (control) (A, B), with amphotericin B (C, D), and with ketoconazole (E, F). (M) Molecular weight ladder. Sizes expected for the amplified fragments were 49 bp and 188 bp for nonretention or retention of the intron, respectively. (II) Schematic overview of intron‐3 retention, as compared to the genomic DNA and mRNA organization of the *asn‐2* gene of *N. crassa*.

**Figure 4:**
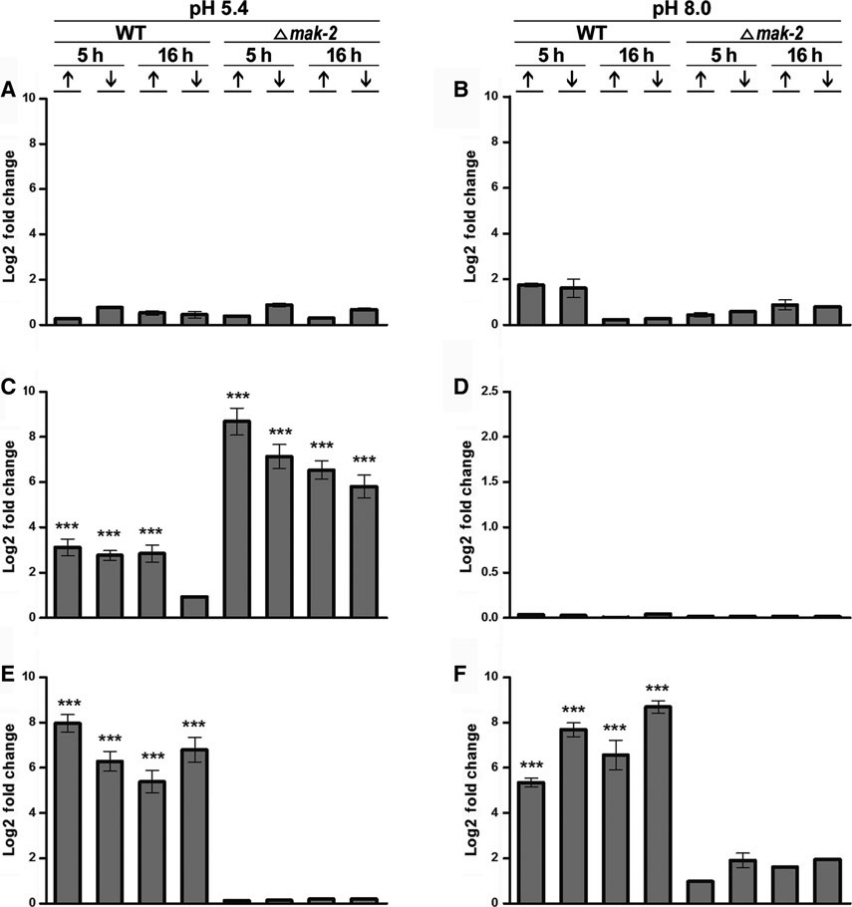
Real‐time PCR (qRT‐PCR) validation of intron‐3 transcript levels in the *asn‐2* gene in *N. crassa*. Strains Δ*mak‐2* mutant and St.L.74A were incubated for 5 h and 16 h in high‐ (10 mm) (↑) or low‐Pi (100 μm) (↓), at pH 5.4 and pH 8.0, in the absence of antifungal (control) (A, B), with amphotericin B (C, D), and with ketoconazole (E, F). qRT‐PCR data are representative of the average values ± standard deviation (SD) obtained from three independent experiments. Statistically significant values are indicated by asterisks: Tukey's *ad hoc* test, ***P < 001.

**Figure 5:**
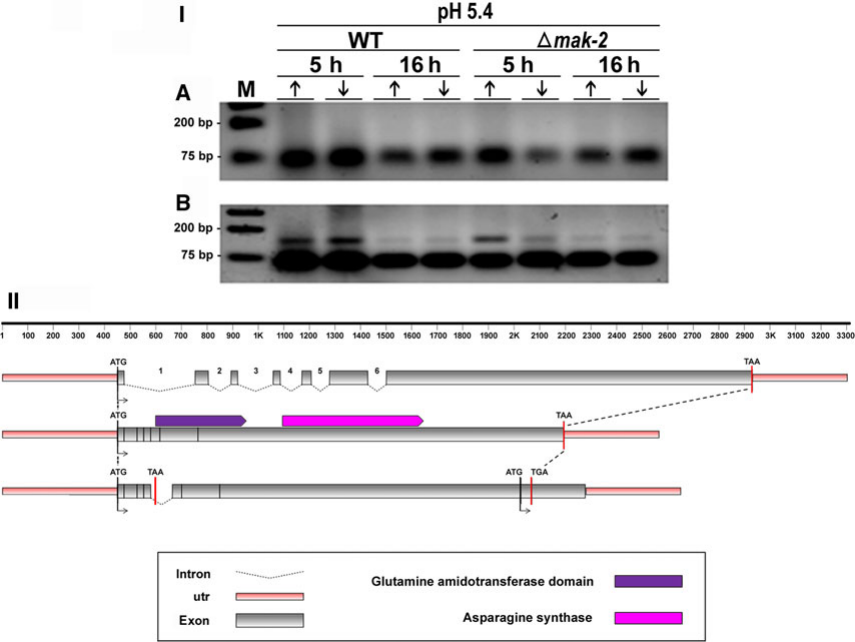
(I) Retention of intron‐4 visualized by RT‐PCR during pre‐mRNA processing of the *asn‐2* gene in *N. crassa*. Strains Δ*mak‐2* mutant and St.L.74A were incubated for 5 h and 16 h in high‐ (10 mm) (↑) or low‐Pi (100 μm) (↓) at pH 5.4 in the absence (A) and the presence of ketoconazole (B). (M) Molecular weight ladder. Sizes expected for the amplified fragments are 57 and 142 bp for nonretention or retention of the intron, respectively. (II) Schematic overview of intron‐4 retention, as compared to the genomic DNA and mRNA organization of the *asn‐2* gene of *N. crassa*.

**Figure 6:**
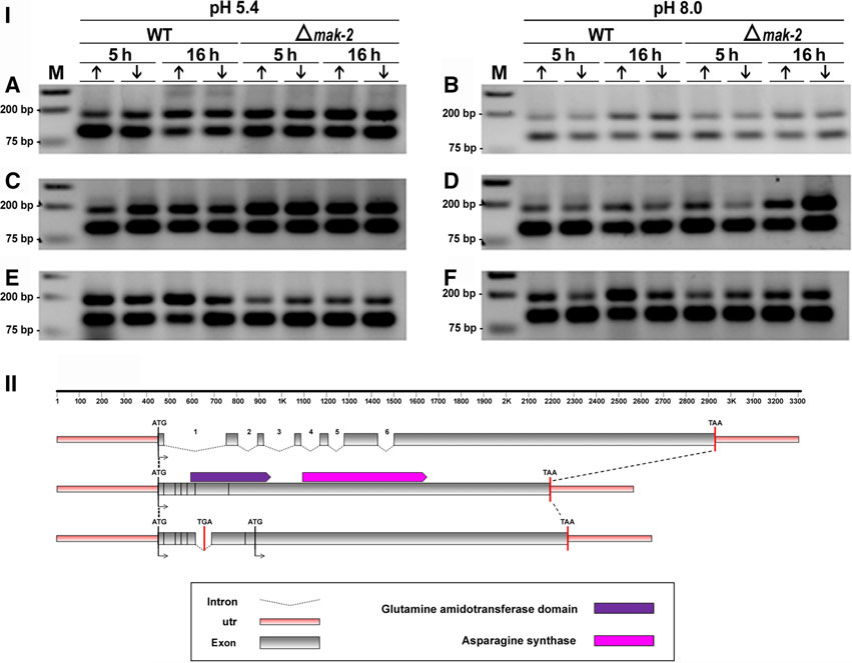
(I) Retention of intron‐5 visualized by RT‐PCR during pre‐mRNA processing *of the asn‐2* gene in *N. crassa*. Strains Δ*mak‐2* mutant and St.L.74A were incubated for 5 h and 16 h in high‐ (10 mm) (↑) or low‐Pi (100 μm) (↓) at pH 5.4 and pH 8.0 in the absence of antifungal (A, B), with amphotericin B (C, D), and with ketoconazole (E, F). (M) Molecular weight ladder. Sizes expected for the amplified fragments are 112 and 182 bp for nonretention or retention of the intron, respectively. (II) Schematic overview of intron‐5 retention, as compared to the genomic DNA and mRNA organization of the *asn‐2* gene of *N. crassa*.

Prediction of putative intron retention in these three genes using publicly available RNAseq data showed that different introns in the same gene might undergo differential splicing depending on the experimental conditions (Fig. 2). Our experimental confirmation (Figs 3, 4, 5, 6) of the retention of some of these predicted introns is useful since it allows the identification of alternative splicing events by using the next‐generation RNA sequencing technologies.

### Genes and proteins

#### Asparagine synthetase 2

Asparagine is biosynthesized in eukaryotes from l‐aspartate using glutamine as the amino group donor, which is an enzymatic reaction driven by the hydrolysis of ATP 30. Asparagine synthetase 2, encoded by the *asn‐*2 gene, catalyzes this metabolic pathway. However, in some organisms such as *Escherichia coli*, a second metabolic pathway utilizes either ammonia or glutamine as the amino group donor 30. Little is known about the roles of asparagine, glutamate, glutamine, and aspartate, aside from being important sources for the *de novo* biosynthesis of nucleotides and various amino acids. However, asparagine may act as a metabolic regulator of cellular adaptation to glutamine depletion likely by supporting the availability of TCA cycle intermediates and the supply of reduced nitrogen needed to maintain the synthesis of nonessential amino acids. Additionally, glutamine derivatives have been proposed to be an essential component of glutamine‐dependent cell survival in mammals. Thus, asparagine synthetase‐2 may play a regulatory role in the intricate metabolism of nitrogen and carbon in eukaryotes 32. In *N. crassa*, transcription of the *asn‐2* gene is up‐regulated in cultures with abundant Pi, a physiological condition where the transcriptional regulator NUC‐1 is nonfunctional. Thus, the *asn‐2* gene is not Pi‐repressible. We also provided conclusive evidence that transcription of Pi‐repressible genes is down‐regulated in the Δ*mak‐2* strain, irrespective of the supply of Pi, whereas, for some non‐Pi‐repressible genes, this effect is not observed 19. Interestingly, another *asn* gene, coding for an asparagine synthetase domain‐containing protein 1, here renamed *asn‐1* (KEGG: 07300.7), is also not Pi‐repressible 19.

Pre‐mRNA processing of the *asn‐2* gene of *N. crassa* (KEGG: NCU04303) apparently responds to different effectors, depending on the intron being considered (Figs 3, 4, 5, 6). Six putative introns were identified in the *asn‐2* gene and were referred to as introns 1– 6, based on their 5′ to 3′ locations (http://www.broadinstitute.org/annotation/genome/neurospora/MultiHome.html). The retention of intron‐3 is weak at both pH 5.4 and pH 8.0 in the absence of antifungal drugs (Fig. 3IA,B), which is in agreement with the RNA‐seq analysis (Fig. 2). In the presence of amphotericin B, however, retention increases at pH 5.4 but not at pH 8.0 (Fig. 3IC,D). In the presence of ketoconazole, retention of intron‐3 increases at pH 5.4 and pH 8.0 in the 74A strain, but not in the ∆*mak‐2* strain (Fig. 3IE,F). These observations were confirmed by qRT‐PCR analysis (Fig. 4), which indicated that these antifungal drugs indeed affect the transcriptional process in *N. crassa*. Schematic representation of intron‐3 retention, as compared to the genomic DNA and mRNA organization of the *asn‐2* gene of *N. crassa*, showed disruption of the asparagine synthase and glutamine amidotransferase domains of the protein leading to a probable inactivation of the catalytic activities of this enzyme. It is also observed a nucleotide long sequence, probably not translated (Fig. 3II).

Intron‐4 was consistently removed from the *asn‐2* transcripts in the absence of antifungal drugs at both pH 5.4 and 8.0. Interestingly, intron‐4 was retained in both 74A, and Δ*mak‐2* strains cultured for 5h in the presence of ketoconazole at pH 5.4, irrespective of current Pi changes (Fig. 5I) but was spliced out at pH 8.0 (not shown) in both wild‐type and Δ*mak‐2* strains. Our prediction of intron‐4 retention (Fig. 2) in the absence of antifungal drugs was not confirmed by our data (Fig. 5I). However, retention of this intron may be affected by the different composition of the culture medium used for the RNAseq experiments, as occurred in the antifungal treatment (Fig. 5I). Schematic representation of intron‐4 retention, as compared to the genomic DNA and mRNA organization of the *asn‐2* gene of *N. crassa*, showed disruption of the asparagine synthase and glutamine amidotransferase domains of the protein leading to a probable inactivation of both catalytic activities of this enzyme. It also showed a nucleotide long sequence which was probably not translated (Fig. 5II).

The retention of intron‐5 in the *asn‐2* transcripts was observed in all culture conditions tested, irrespective of the absence or presence of antifungal, levels of extracellular Pi, strains assayed, or incubation time (Fig. 6I), confirming our predicted results. However, it is clear that retention of intron‐5 is lower at pH 8.0 in the absence of antifungal compared to pH 5.4 (Fig. 6I). Schematic representation of intron‐5 retention, as compared to the genomic DNA and mRNA organization of the *asn‐2* gene of *N. crassa* (Fig. 6I), showed disruption of the glutamine amidotransferase domain of the protein leading to a probable inactivation of the catalytic activity of this enzyme. However, retention of intron‐5 led to a nucleotide long sequence in which the sequence of the asparagine synthase domain seems conserved. This sequence defines a putative protein possibly capable of retaining this enzymatic activity (Fig. 6II).

#### C6‐zinc finger regulator (*fluffy*) (KEGG: NCU08726)

**Figure 7:**
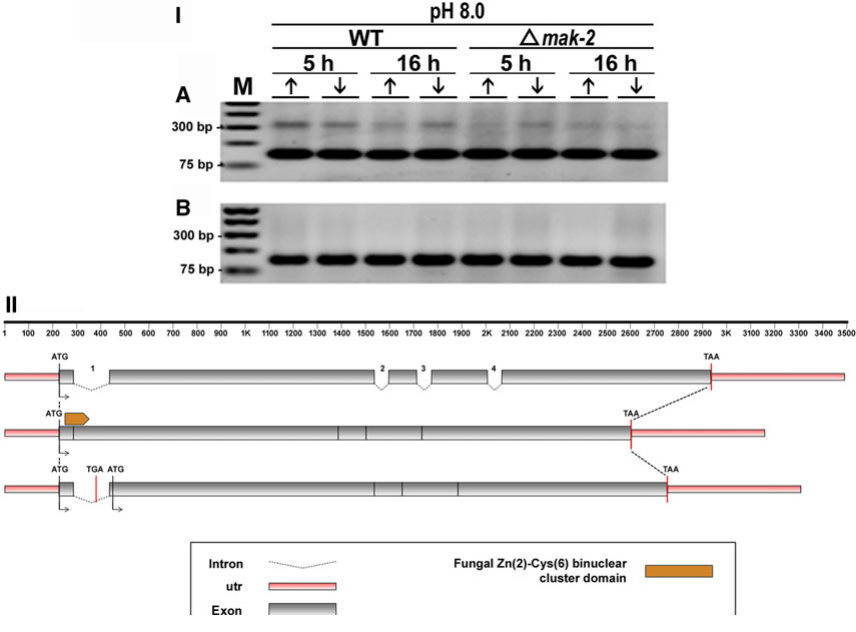
(I) Retention of intron‐1 visualized by RT‐PCR during pre‐mRNA processing of the *fluffy* gene in *N. crassa*. Strains Δ*mak‐2* mutant and St.L.74A were incubated for 5 h and 16 h in high‐ (10 mm) (↑) or low‐Pi (100 μm) (↓) at pH 8.0 in the absence (A) and presence (B) of ketoconazole. (M) Molecular weight ladder. Sizes expected for the amplified fragments are 149 and 299 bp for nonretention or retention of the intron, respectively. (II) Schematic overview of intron‐1 retention, as compared to the genomic DNA and mRNA organization of the *fluffy* gene of *N. crassa*.

The *fluffy* gene of *N. crassa* (NCU08726) is a transcription factor that directly regulates two of the five genes involved in cellular conidiation 33, 34, 35. Four putative introns were identified in the fluffy gene and were referred to as introns 1–4, in 5′ to 3′ order of appearance. We correctly predicted the retention of intron‐1 in the RNAseq experiments at pH 8.0 in the absence of antifungal drugs. Interestingly, retention was not observed in any other culture conditions assayed (Fig. 7I, and results not shown). The schematic representation of intron‐1 retention, as compared to the genomic DNA and mRNA organization of the *fluffy* gene of *N. crassa*, showed disruption of its DNA‐binding domain leading to a predicted inactive protein. Also, retention of intron‐1 led to a nucleotide long sequence that defines a putative protein in which the zinc‐finger domain is absent (Fig. 7II).

#### CaaX farnesyltransferase beta subunit Ram1 (*ram‐1*) (KEGG: NCU05999)

**Figure 8:**
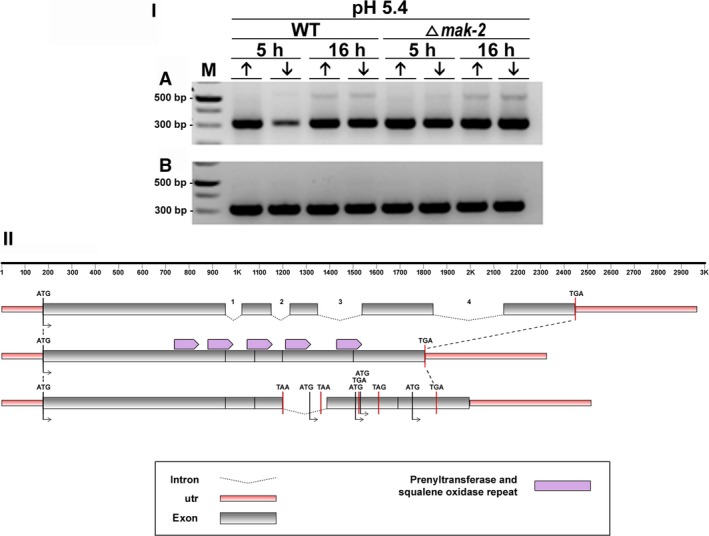
(I) Retention of intron‐3 visualized by RT‐PCR during pre‐mRNA processing of the gene coding for farnesyl transferase beta subunit Ram1 in *N. crassa*. Strains Δ*mak‐2* mutant and St.L.74A were incubated for 5 h and 16 h in high‐ (10 mm) (↑) or low‐Pi (100 μm) (↓) at pH 5.4 in the absence (A) and presence (B) of ketoconazole. (M) Molecular weight ladder. Sizes expected for the amplified fragments are 313 and 503 bp for nonretention or retention of the intron, respectively. (II) Schematic overview of intron‐3 retention, as compared to the genomic DNA and mRNA organization of the gene coding for a farnesyltransferase (beta subunit Ram1) of *N. crassa*.

The *ram*‐1 gene of *N. crassa* (NCU05999) codes for a farnesyltransferase, an enzyme that catalyzes the addition of farnesyl pyrophosphate to a cysteine of a G protein, resulting in a farnesylated protein and a diphosphate. G proteins play crucial roles in many signaling processes 7, 36, 37. Thus, the proper functioning of this farnesyltransferase is likely necessary for signal transduction in eukaryotic microorganisms. The catalytic domain of the prenyltransferase‐like activity of this enzyme is harbored in the N‐terminal domain, whereas the catalytic domain of the prenyltransferase and squalene oxidase activities is repeated over the protein body (http://www.broadinstitute.org/annotation/genome/neurospora/MultiHome.html). Four putative introns were identified in the *ram*‐1 gene. The retention of intron‐3 was observed only at pH 5.4 in both wild‐type and ∆*mak‐2* strains cultured for 16 h and in the absence of antifungal drugs (Fig. 8I). The schematic representation of intron‐3 retention, as compared to the genomic DNA and mRNA organization of the *ram‐1* gene of *N. crassa*, showed disruption of its catalytic repeat domain leading to a predicted inactive protein (Fig. 8II).

## Conclusions

Intron retention is one of the five top types of alternative splicing in eukaryotic microorganisms that are represented by alternate 5′ or 3′ splice sites. The splicing of numerous genes is modulated in response to nutrient signaling, including many cellular stressors. Here, we show that intron retention in the genes encoding asparagine synthetase 2 (*asn‐2*), C6‐zinc finger transcription factor (*fluffy*), and a farnesyltransferase (*ram‐1*) is modulated by antifungal drugs. In general, the assayed antifungal promoted the disruption of the structural domains of these proteins probably leading to their inactivation. Antifungal‐induced intron retention in some cases is dependent on MAPK protein MAK‐2. Furthermore, we showed that putative introns in the same gene can undergo differential splicing, and confirmed by RT‐PCR experiments the differential retention of introns predicted by RNAseq analyses. Intron retention in *N. crassa*, and probably in other fungi, is an adaptive genetic response to the toxic effect of antifungal. The antifungal transduction signaling pathway comprises, besides the regulation of mRNA processing in many genes, also the spliceosomal machinery, which may affect the whole fungal metabolism.

Our findings provide an overview of the broad spectrum of metabolic and cellular activities that can be affected by antifungals outside of the inhibition of enzymes of the ergosterol biosynthetic pathway. Thus, antifungals can affect many cellular processes through differential intron retention, leading to changes in gene transcription and the rate of transcript synthesis or degradation, thereby influencing recruitment of many cellular components such as proteins, nucleic acids, and by‐products. These results emphasize the complexity of the metabolic modulation exerted by antifungal signaling.

## Author contributions

AR and NMM‐R conceived and designed the project, supervised the research study, and prepared the manuscript. NSM and PMS acquired the data. RS‐R performed the bioinformatics analyses. AR, NSM, PMS, RS‐R, and NMM‐R analyzed and interpreted the data.
